# Benchmarking untargeted metabolomics data quality with allopurinol-induced perturbations

**DOI:** 10.1007/s11306-026-02457-x

**Published:** 2026-05-16

**Authors:** Terje Vasskog, Pia J. Heinsvig, Ekaterina Sharashova, Terkel Hansen, Torbjørn N. Myhre, Marie Mardal

**Affiliations:** 1 Natural Products and Medicinal Chemistry Research Group, Department of Pharmacy, UiT - The Arctic University of Norway, Hansine Hansens veg 18, 9019 Tromsø, Norway; 2 Section of Forensic Chemistry, Department of Forensic Medicine, University of Copenhagen, Copenhagen, Denmark; 3 Department of Community Medicine, UiT - The Arctic University of Norway, Tromsø, Norway; 4 Biotechnology and Nanomedicine, SINTEF Industry, Trondheim, Norway

## Abstract

**Introduction:**

We present a simple test to assess whether a metabolomics dataset is fit-for-purpose. Current qualitycontrol approaches do not directly evaluate the ability to recover biologically meaningful perturbations.

**Objectives:**

To evaluate whether known drug-induced metabolic perturbations can serve as internal benchmarks fordataset quality.

**Methods:**

In a study (the TROMBOLOME study, unrelated to allopurinol therapy), 1,000 serum samples were analyzedwith one targeted and two untargeted metabo lomics panels. Samples were classified as allopurinol-positive (N=19)using detection of allopurinol analytical targets. Endogenous metabolite markers of allopurinol therapy wereevaluated based on hypotheses derived from the literature. Statistical evaluation was performed using Mann–Whitney U-tests.

**Results:**

The hypothesis of upregulation was supported for xanthine, orotate, and orotidine (p < 0.0001) inallopurinol-positive cases (N = 19). These findings demonstrate repro ducibility of well-characterized metabolicperturbations within the dataset.

**Conclusion:**

In the absence of external quality assessment schemes for untargeted metabolomics, such benchmarkscould provide a practical way to evaluate whether datasets are suitable for downstream biological interpretation.The proposed targeted exposomics approach complements traditional QC metrics by assessing biologicalrecoverability.

**Supplementary Information:**

The online version contains supplementary material available at 10.1007/s11306-026-02457-x.

## Introduction

Metabolomics studies often seek to uncover molecular mechanisms underlying disease development and progression. Understanding these complex patterns is essential for advancing pathophysiology, which in turn may lead to new treatment or preventive strategies. Progress in health sciences and metabolomics requires trust in existing research, enabling researchers to build on previous findings. To achieve this, researchers must ensure and justify that the study designs are feasible and that acquired datasets are of sufficient quality to properly evaluate hypotheses. In addition, studies must be described and defined in sufficient detail to ensure reproducibility.

Benchmarking the quality of data is essential before presenting results. This assurance typically comes when bioanalytical procedures successfully pass external quality assessment (EQA), which is currently not available for untargeted metabolomics. Recommended metabolomics quality control focuses on the evaluation of intrinsic properties of a dataset, such as pooled Quality Control (QC)_pool_ variance, within-run linearity studies, and benchmarking identifications against Standard Reference Material (SRM) 1950 (Sumner et al., 2007; Broadhurst et al., 2018). These steps provide valuable information on technical variance and performance. However, these methods only infer if the dataset is fit-for-purpose, given that the purpose is to find biologically relevant metabolic perturbations.

We therefore propose using a targeted exposomics approach to verify the quality of untargeted metabolomics data sets. We here define targeted exposomics as the investigation of predefined perturbations of endogenous markers caused by an exogenous molecule (here: the drug allopurinol). The perturbations should have a well-documented, clear mechanistic origin and direction. This is based on the premise that when well-documented metabolic perturbations are reproduced in a new dataset, then that dataset should be fit for metabolomics data analysis. Passing such a benchmark test enhances confidence in results from the evaluated dataset.

**Fig. 1:**
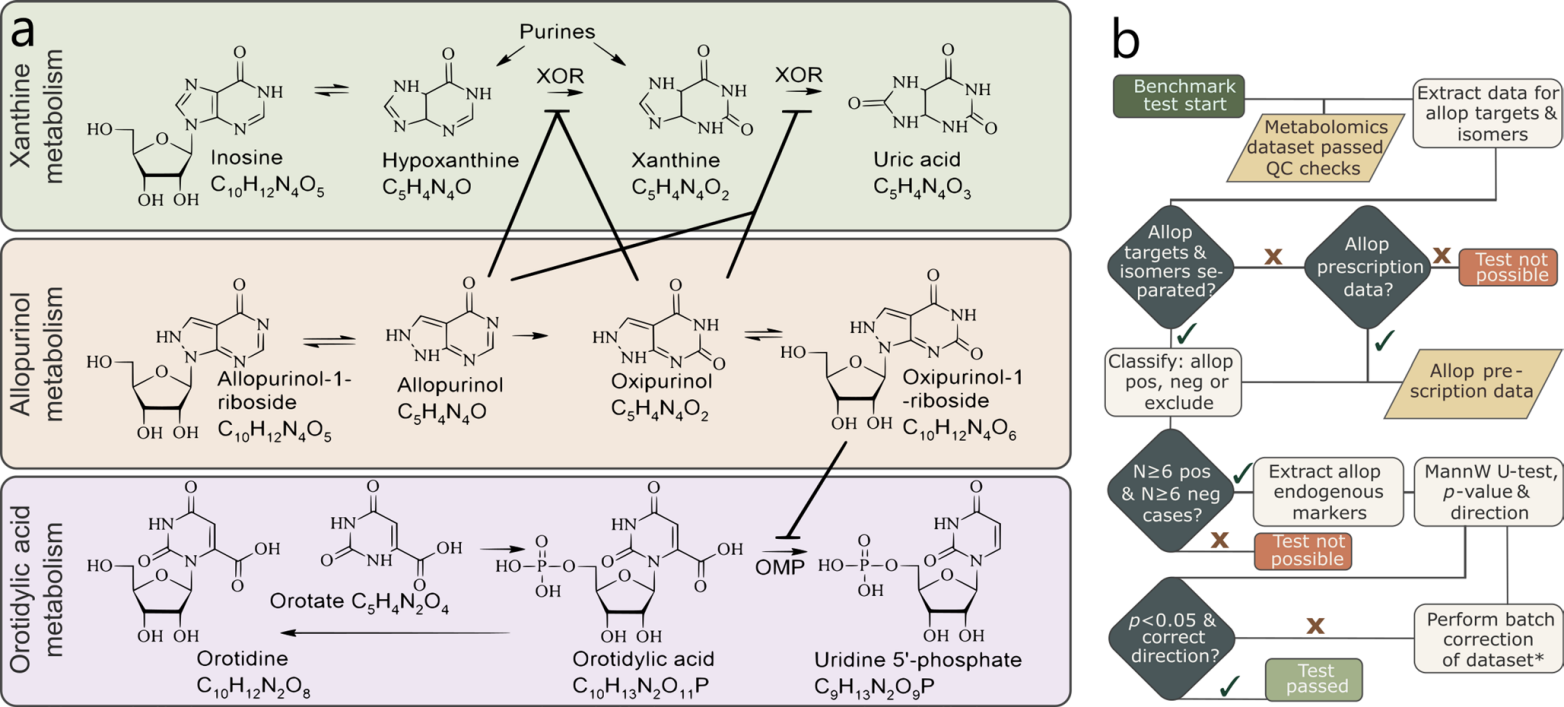
**a** Metabolic pathways for xanthine metabolism (green), allopurinol metabolism (sand), and orotidylic acid metabolism (lavender), where a solid line represents enzyme inhibition of xanthine oxidoreductase (XOR) or orotidine monophosphate decarboxylase (OMP), **b** Flowchart of the allopurinol benchmark test. Allopurinol (Allop), Quality Control (QC), Mann-Whitney (MannW), positive (pos), negative (neg). *: Only perform batch corrections that will be used in later data analysis

Allopurinol and its active metabolite oxipurinol inhibit xanthine oxidoreductase (XOR), thereby reducing uric acid production (Day et al., 2007; Rundles, 1985) (Fig. 1a). This inhibition results in accumulation of the uric acid precursors, xanthine and hypoxanthine (Rundles, 1985; Sun et al., 2022). The riboside metabolite of oxipurinol also inhibits orotidine monophosphate decarboxylase (Hauser et al., 1990) which converts orotidylic acid to uridine-5’-phosphate (Fig. 1a) that leads to increased excretion of orotidine and orotate (Hauser et al., 1990). In 2023, the prescription rate of allopurinol in Norway was 5 defined daily doses per 1,000 inhabitants (Folkehelseinstituttet, 2024). Accordingly, cross-sectional metabolomics datasets from comparable countries are expected to include sufficient allopurinol-positive cases for this benchmark test.

The proposed benchmark evaluates biological recoverability and does not replace or proxy traditional QC metrics for global analytical performance. To demonstrate the utility of the tests, we use a dataset comprising metabolite profiles from 1,000 serum samples analyzed with one targeted and two untargeted metabolomics methods. This targeted exposomics test evaluates perturbations of endogenous metabolites induced by allopurinol (flowchart in Fig. 1b).

## Methods

The data set used for the present study was acquired for a case-cohort study, designed to identify risk markers for the development of first-time acute myocardial infarction. A total of 1,000 serum samples had been analyzed with one targeted and two untargeted metabolomics methods. A more detailed description of the samples and instrumentation is available in supplementary information (SI).

### Study samples

Study samples were collected as part of the seventh survey of the Tromsø Study in 2015–2016, as presented elsewhere (Hopstock et al., 2022). Aliquots of collected serum samples had been stored at −80 °C. Samples were analyzed sequentially following the Tromsø study number system. Study subjects were selected for a case-cohort study which does not translate to a systematic bias toward allopurinol cases. Information on the preparation of QC samples is available in SI.

### Analytical methods

*Targeted metabolomics* with the Biocrates MxP^®^ Quant 500 kit was prepared and analyzed according to the manufacturer’s instructions (Zararsiz et al., 2024). The kits were analyzed on a Waters TQ-XS coupled with a Waters I-class UPLC (Waters Corp, Milford, USA), with two liquid chromatography – tandem mass spectrometry (LC-MS/MS) and two flow-injection analysis (FIA)-MS/MS methods per sample (Sommer et al., 2019).

*Untargeted metabolomics* analyses were based on previously presented and validated methods (Grijseels et al., 2024). Serum samples were prepared on a Tecan Fluent 780 liquid handler (Tecan, Männedorf, Switzerland) using precipitation with acetonitrile: methanol (75:25, v/v %) spiked with internal standards (ISTDs). The hydrophilic interaction liquid chromatography (HILIC) aliquots were directly injected on the LC-MS, whereas the evaporated reversed-phase LC (RPLC) plates were stored at −20 °C and resuspended before injection. Analyses were performed with a Vanquish Horizon UHPLC interfaced with an Orbitrap ID-X Tribrid Mass Spectrometer (Thermo Scientific, Waltham, MA). Molecules separated by the HILIC and RPLC methods were analyzed with negative and positive electrospray ionization, respectively. Ammonium acetate buffer (pH 9.0) was used for the HILIC method, and ammonium formate (pH 3.0) was used for the RPLC method. ACQUITY BEH amide (100 × 2.1 mm, 1.7 μm) and ACQUITY HSS T3 (150 × 2.1 mm, 1.8 μm) analytical columns were purchased from Waters. Data for every sample were acquired in fullMS using the Orbitrap analyzer and data-dependent MS/MS was acquired with the ion trap analyzer with one scan of each per cycle.

### Analytical runs and quality control

The serum samples were assigned to 13 targeted metabolomics batches and 23 untargeted metabolomics batches. The untargeted batches each contained 44 study samples, 12 QC_pools_, two sets of levelled QCs (QC_L_ and QC_H_), a QC_pool_ without ISTD, SRM1950, method blanks with and without ISTD, and a QC_batchpool_. QC_pools_ were injected at least every 7 injections. A sample list that displays batch structure is presented in Table SI1, inspired by Roberts et al. (2022).

System suitability was assessed using a system control solution containing representative metabolites, analyzed together with system blanks prior to batch acquisition. Acceptance criteria included mass accuracy within ± 5 ppm without lock-mass correction and ± 2 ppm with lock-mass correction, retention time stability within ± 0.3 min (HILIC) or ± 0.1 min (RPLC), and minimum signal intensity thresholds. During acquisition, sample release was based on continuous monitoring of internal standards to verify injection performance, sensitivity stability, and retention time consistency. If internal standard signal intensity decreased below approximately 50% relative to post-maintenance performance, system suitability was re-evaluated and corrective maintenance performed if required. Analytical run release was evaluated using predefined endogenous metabolites: acceptance required expected semi-quantitative trends (upregulation in QC_H_ and downregulation in QC_L_ relative to QC_pool_ and absence in blanks). Exact composition of QC materials, sample preparation settings, key acquisition settings (including lock-mass settings) and quality control measures are described in more detail in SI.

### Data analysis

Acquisition for the targeted metabolomics was performed with MassLynx (Waters), and data analysis was performed in WebIDQ (Biocrates, Innsbruck, Austria). Batches were QC-normalized based on the median of a QC run four times per batch, and results were exported in µM. Data acquisition for the untargeted metabolomics was performed in Xcalibur (Thermo) and both quality control and data analysis were performed with Skyline (MacLean et al., 2010) and Freestyle 1.8 (Thermo). A list of analytical drug targets was compiled consisting of allopurinol and its main circulating metabolites together with endogenous metabolite markers. Chromatographic peak areas were extracted with Skyline (MacLean et al., 2010). If the extracted ion chromatogram (EIC) area was zero, then the value was imputed with a blank sample average; alternatively the lowest measured value of the given metabolite.

*Compound identification in untargeted metabolomics*: A compound was identified with a precursor ion [M + H]^+^ or [M-H]^−^ in full MS with a mass error below 3 ppm, and an MS/MS ion trap spectrum matching an online reference spectrum, if available. Some metabolites were available in a local library previously made with injected reference standards on the same method (Grijseels et al., 2024), these were labeled as level 1 identifications if the RT error was within 0.3 min–0.1 min for HILIC and RPLC, respectively. If the metabolites were not in the local library, these were identified at level 2 or 3 (Sumner et al., 2007).

A sample was categorized as allopurinol-positive when at least two allopurinol analytical drug targets were identified in a sample. If only one drug target was present, then this sample was removed from the dataset. Absence was defined as a peak area less than 5x blank signal. The samples were also tested for targets of probenecid and febuxostat, which are drugs with the same indication as allopurinol, but with other mechanisms of action. Excel (Microsoft, Redmond, USA), Chemsketch (ACD labs, Toronto, CA), and Python, including the SciPy library (Virtanen et al., 2020), were used for statistics and to produce figures. Allopurinol analytical targets and endogenous metabolite markers were evaluated for statistical significance based on their peak areas. Markers from each of the three metabolomics methods were divided into allopurinol-positive and -negative cases and evaluated using the non-parametric Mann-Whitney U-test, either one- or two-sided based on perturbations reported in the literature.

### Rationale for statistical tests

Sun et al. reported significant upregulation of xanthine and orotate but no significant changes in hypoxanthine levels in serum samples following allopurinol administration (Sun et al., 2022). Studies have reported the increased levels of hypoxanthine and xanthine as the short-term result of allopurinol administration, with only the xanthine elevation being consistently significant (Turnheim et al., 1999; Kaya et al., 2006). These studies found no significant effect on serum uric acid (Sun et al., 2022; Turnheim et al., 1999; Kaya et al., 2006). The elevated urinary levels of orotidine have been reported (Hauser et al., 1990), as an allopurinol challenge test has been used to diagnose ornithine carbamoyl transferase deficiency (Lichter-Konecki et al. 1993). We hypothesized this metabolite to be upregulated (one-sided). The evaluated test was thus one-sided for xanthine, orotate, and orotidine, and two-sided for hypoxanthine and uric acid.

## Results and discussion

**Fig. 2:**
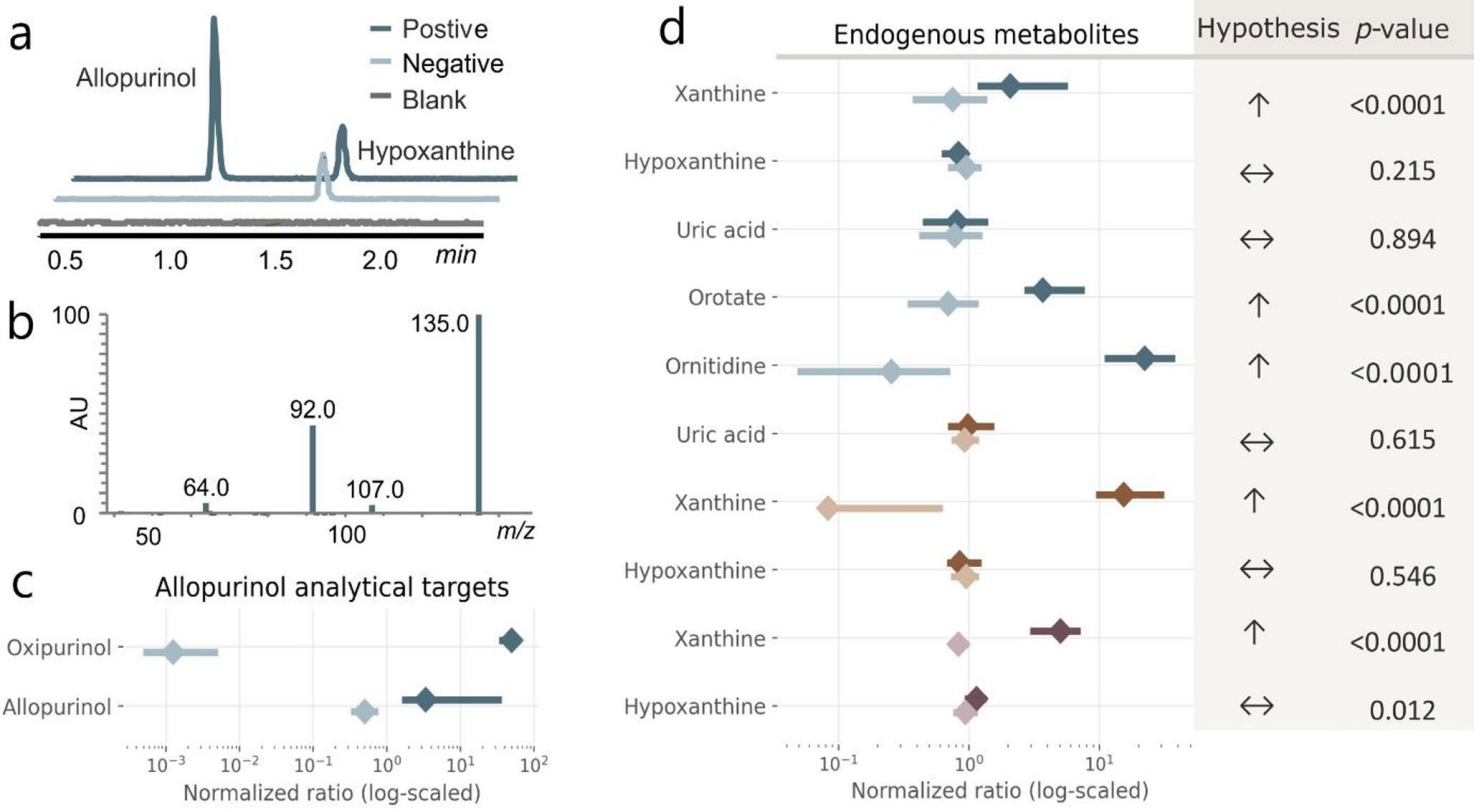
**a** Extracted ion chromatograms (± 3 ppm) from the hydrophilic interaction liquid chromatography – high-resolution mass spectrometry (HILIC-HRMS) method for an allopurinol-positive case (dark blue), allopurinol-negative (light blue), and a blank sample (gray) showing baseline separation between the isomers, **b** Ion trap tandem mass spectrum of allopurinol in an allopurinol-positive case, **c** and **d** paired forest plots of evaluated molecules in allopurinol-positive cases (*N* = 19) in darker colors and -negative cases (*N* = 978) in lighter colors, normalized to the average for each analyte per method. Results are given as median (diamond) and lower and upper interquartile range (line) with the HILIC (blue); Reversed-phase liquid chromatography (RPLC) (orange) or targeted methods (plum). To the right of **d** are the tested hypothesis and corresponding p-value for a one- or two-tailed Mann Whitney U-test with arrows indicating tested hypothesis

For the targeted exposomics test, samples were first categorized as allopurinol-positive or allopurinol-negative and invalid samples were excluded. Baseline separation of the allopurinol/hypoxanthine and oxipurinol/xanthine isomer pairs was achieved with the HILIC method (Fig. 2a), which was therefore used to classify samples. Of the 1,000 samples, 19 were positive for allopurinol and 978 were negative. One sample was excluded due to the detection of febuxostat (see Table SI2), and two were excluded because only one analytical drug target for allopurinol was detected. Forest plots of allopurinol analytical targets are presented in Fig. 2c. Seven endogenous metabolic markers were evaluated; five were reliably detected, with EICs from a positive, negative, and a method blank sample and MS/MS presented in Table SI2.

In our dataset, the endogenous metabolite markers xanthine, orotate, and orotidine were significantly upregulated in allopurinol-positive cases (Fig. 2d). Hypoxanthine appeared upregulated in the quantitative method, but not the two untargeted methods, and this may reflect the lack of selectivity between hypoxanthine and allopurinol. No significant changes in uric acid were observed across methods. The metabolites with predefined directional hypotheses (xanthine, orotate, and ornitidine) show consistent shifts in the expected direction with fold changes ranging from 2.8 to 87.3 for xanthine and ornitidine, respectively, in HILIC, when disregarding metabolites not baseline separated from analytical targets. The remaining metabolites display smaller and less consistent changes. The HILIC method was evidently best suited for the exposomics benchmark we proposed here, since it enabled detection of more relevant metabolites. If laboratories using other methods are interested in using this test, it may well be possible also with other chromatographic methods, since xanthines can be detected with a range of conditions (Grijseels et al., 2024; Talik et al., 2012).

A concordance test can complement the proposed benchmark by revealing laboratory errors and systematic bias. Figure SI1 shows a concordance analysis using the non-parametric Spearman’s rho for methionine detected across all three analytical methods (Spearman’s rho: 0.88–0.92), where high concordance indicates the absence of systematic bias and an adequate dynamic range for the evaluated metabolite.

The tests proposed here require some conditions to be met. For the allopurinol test outlined in the flowchart in Fig. 1b, the samples must be classified, which ideally is achieved with baseline separation of allopurinol targets and its isomers on at least one method. The metabolite markers included in the evaluation must be selected from those that are reliably detectable in the evaluated datasets. A key advantage is that the test does not necessarily require allopurinol prescription data, making it suitable for core facilities and analysts without access to patient records. The test can also be applied to datasets in repositories or marketplaces, to assess data quality. However, prescription data can be used for classification if there is no baseline separation of allopurinol targets and its isomers. We propose using this test with six or more cases in each group to allow sufficient representation of each class for stable evaluation of the metrics. If a dataset fails this benchmark test using raw peak areas, batch correction may be applied and the statistical analysis repeated.

## Conclusions

In summary, we present a simple benchmark test for assessing the quality of metabolomics datasets based on the recovery of well-characterized metabolic perturbations induced by allopurinol. The test does not replace or proxy traditional QC metrics for global analytical performance. In the absence of commercial EQA schemes, this approach could provide a practical means to evaluate whether existing datasets are fit-for-purpose.

## Supplementary Information

Below is the link to the electronic supplementary material.

Supplementary Material 1

## Data Availability

No datasets were generated or analysed during the current study.
