# Supplementary material for: Benchmarking untargeted metabolomics data quality with allopurinol-induced perturbations
Source: Metabolomics. 2026 May 16;22(3):74. doi: 10.1007/s11306-026-02457-x (PMC13179923; doi:10.1007/s11306-026-02457-x)
Supplement: Supplementary file 1 — Supplementary Material 1 [file 11306_2026_2457_MOESM1_ESM.docx]

Supplementary information for: Benchmarking Untargeted Metabolomics Data Quality with Allopurinol-Induced Perturbations

Terje Vasskog^a^, Pia J. Heinsvig^b^, Ekaterina Sharashova^c^, Terkel Hansen^a,d^, Torbjørn N. Myhre^a^, Marie Mardal^a,b*^

^a^ Natural Products and Medicinal Chemistry Research Group, Department of Pharmacy, UiT - The Arctic University of Norway, Tromsø, Norway

^b^ Section of Forensic Chemistry, Department of Forensic Medicine, University of Copenhagen, Copenhagen, Denmark

^c^ Department of Community Medicine, UiT - The Arctic University of Norway, Tromsø, Norway

^d^ Biotechnology and Nanomedicine, SINTEF Industry, Trondheim, Norway

Corresponding Author

* Marie Mardal

Marie.mardal@uit.no, +47 40859756

Department of Pharmacy

The Arctic University of Norway

Hansine Hansens veg 18

9019 Tromsø

Norway

1. **Materials and methods:**

**1.1 Standards and reagents**

Acetonitrile, methanol, isopropanol, ammonium formate, ammonium acetate, formic acid, and sodium hydroxide of LC-MS grade and ethanol of gradient grade were purchased from Fisher Scientific (Pittsburg, USA) or Honeywell (Charlotte, USA). The mass spectrometry metabolite library (MSMLS 230-01) panel was purchased from IROA technologies (Sea Girt, USA). SRM 1950 and Internal standards (ISTD): Acetyl-L-carnitine-D3, Hexadecanoyl(palmitoyl)-L-carnitine-D3, L-Leucine-5,5,5-D3, L-Tryptophan-(indole-D5), L-Methionine-(methyl-D3), Stearic acid-18,18,18-D3, Chenodeoxycholic-2,2,3,4,4,6,6,7,8-D9 acid, 18:0-D35 Lyso PC and other chemicals and reagents were purchased from Merck Life Science (Darmstadt, Germany).

Biocrates MxP Quant 500 kits were purchased from Biocrates AG (Innsbruck. Austria).

**1.2 QC samples**

*Untargeted metabolomics*

Serum for quality control (QC) samples were obtained from 54 blood donors (26 female and 27 male) from the University Hospital of Northern Norway. The serum was prepared by centrifuging the individual blood samples at 1500 g for 10 min at 4 °C. A QC_pool_ sample was prepared by mixing serum from individual blood donors in a large batch. The QC_noIS_ sample was prepared as the QC_pool_ without the addition of ISTD. Method blank samples were phosphate-buffered saline, one prepared as sample and the other prepared without ISTD. Leveled QC samples, a QC high (QC_H_) and a QC low (QC_L_), were prepared by freeze-drying six mL QC_pool_ for 48 hours at -50°C and 0.05 mBar. Then, the QC_H_ was prepared by reconstituting the precipitate with 4 mL LC-MS grade water. QC_L_ was prepared by diluting 1 mL QC_H_ with 2 mL LC-MS grade water. All QC samples were aliquoted into polypropylene tubes and stored at -80 °C for long-term storage. QCs were moved to a -20 °C freezer for easier access during analysis (<4 months).Freeze-and-thaw cycles for all QCs, including SRM 1950, were fixed to two cycles. For every analytical batch, a QC_batchpool_ was produced by volume-proportional mixing of each sample assigned to that batch in a separate cryotube.

*Targeted metabolomics*

QC samples for targeted metabolomics were included in the Biocrates MxP® Quant 500 kit and prepared according to the vendor’s recommendations(1,2).

**1.3 Sample preparation**

Study samples were thawed on ice, and all aliquots were made during one day (one thawing). First, 10 µL ware added to the Biocrates capture plate. Then, the samples were moved to the automated liquid handler, that aliquoted 50 µL into a 96-well plate and 5 µL to a cryotube for QC_batchpool_s. Between transfers the study samples were stored on ice. After the last transfer was made, the study samples were put back in the -80 °C freezer and handed back over to the Tromsø study.

Extraction was performed on a Tecan Fluent 780 liquid handler (Tecan, Männedorf, Switzerland), with the workflow presented previously(3). Fifty µL sample was transferred to a 96-well plate and 450 µL cold ACN:MeOH (75:25, v/v %) either with ISTDs or without ISTD for QC_noIS_ and method blank without ISTD, was added while shaking (BioShake, QInstruments; 1300 RPM). After 3 additional minutes of shaking, the plate was covered by a lid and centrifuged (Hettich universal 320 R) for 12 min at 4 °C and 887 g for pelleting the proteins. In the liquid handler, 50 µL of supernatant was transferred to the hydrophilic interaction liquid chromatography (HILIC) injection plate that was sealed and analysed on the LC-MS instrument described below in section 1.4 for HILIC analyses. For the reversed-phase LC (RPLC) analyses, 300 µL of the supernatant was transferred to another injection plate, evaporated to dryness at 45 °C under a gentle stream of nitrogen (Techne sample concentrator, Antylia Scientific, Vernon Hills, USA), and frozen at -20 °C until analysis. Before RPLC injections, evaporated samples were resuspended by adding 20 µL of water:MeOH (50:50, v/v %), shaking the plate; followed by adding 80 µL LC-MS grade water, and finished by another round of shaking.

The final solution of the ISTDs in the quenching medium ranged from 9 to 222 µg/L, optimized for this application. The selection of ISTD was inspired by Zheng et al(4). All QC samples, except for system suitability and instrument blanks, were prepared as samples with or without ISTD.

**1.4 Instrumentation**

*Untargeted metabolomics* analysis was performed with a Thermo Scientific Vanquish Horizon UHPLC system interfaced with an ID-X Tribrid Mass Spectrometer (Thermo Scientific, Waltham, MA). Two analytical methods were employed based on HILIC or RPLC with negative or positive electrospray ionization (ESI- or ESI+), respectively. The injection volume was 3 µL for all methods. Analytical columns were purchased from Waters Corp (Milford, USA) comprising an ACQUITY BEH amide (100 x 2.1 mm, 1.7 µm) for HILIC analysis, and an ACQUITY HSS T3 (150 x 2.1 mm, 1.8 µm) for RPLC analysis. The flow rate was 0.45 ml/min for HILIC and 0.35 ml/min for RPLC analyses, both maintained at 50 ⁰C. Mobile phases for both HILIC and RPLC were premixed. HILIC mobile phase A consisted of 10 mM ammonium acetate at pH 9 with 10% acetonitrile, and mobile phase B consisted of 10 mM ammonium acetate at pH 9 with 90% acetonitrile. RPLC mobile phase A consisted of a 90:10 v/v% (5 mM ammonium formate adjusted to pH 3.0: acetonitrile with 0.1% formic acid). RPLC mobile phase B consisted of a 5:95 v/v% (5 mM ammonium formate adjusted to pH 3.0: acetonitrile with 0.1% formic acid). The HILIC gradient was as follows. -6-2.5 min (100% A), 2.5-9 min (60% A), 9-9.2 min (60% A). The RPLC gradient was as follows: -0.6-0.5 min (100% A), 0.5-11 (67.5% A), 11-12 (100% B), 12-14 min (100% B), 14-14.5 (0% B). Injections started at 0 min. The autosampler was maintained at 10 ⁰C.

MS settings: Heated Electrospray Ionization source and ion transfer parameters applied were as follows: sheath flow rate (arbitrary units) = 50, auxiliary gas flow rate (arbitrary units) = 10, sweep gas flow rate (arbitrary units) = 1, spray voltage = 3.5 kV (positive) or -2.5 kV (negative), ion transfer tube temperature = 325 °C, vaporizer temperature = 350 °C. For relative quantification, full-scan MS data was acquired in the Orbitrap mass analyser with the following settings: Resolution = 60,000, scan range m/z 70-800, normalized AGC target = 25. The ion trap analyser was used to acquire data-dependent MS/MS scans (one per cycle) on individual samples with the following settings: Isolation mode: quadrupole, isolation window: *m/z* 1.2, activation mode: HCD, collision energy (stepped): 20, 35, 50 %, maximum injection time: 100 ms, normalized AGC target: 25 %, ion trap scan rate: zoom. For the ion trap scans, the following filters were added: Filter precursor selection range: 100-650 m/z (only RPLC), Dynamic exclusion for 4 s (RPLC) or 5 s (HILIC), Filter charge state: include only 1, Apex detection: expected peak width:6 s (RPLC) or 12 s (HILIC). Before every run, lock-mass correction with fluoranthene (Easy-IC) was applied. Furthermore, the QC_Batchpool_ samples were analysed with AcquireX, but these data were not used in this publication.

*Targeted metabolomics* analyses were performed with a Waters TQ-XS (Waters corp, Milford, USA) coupled with a Waters I-class (Waters corp, Milford, USA) UPLC, with two LC-MS/MS and two flow-injection analysis (FIA)-MS/MS methods per sample. For FIA, the autosampler was directly connected to the MS with PEEK tubing. The methods were provided by the vendor.

**1.5 Untargeted metabolomics quality control**

For suitability testing, a system blank and a system control (QC_SS_) containing 0.1 mg/L quercetin, amitriptyline, histidine, arginine, labetalol, doxepin, proline, and tryptophan was analysed on each analytical method. The evaluation was based on retention time, signal intensity, and mass accuracy in full scan MS before and after sample analyses, alongside a system blank consisting of methanol/acetonitrile to identify system contamination (inspecting the total ion chromatogram). The QC_SS_ was run with and without Easy-IC lock-mass correction to monitor mass error. The acceptance criteria were a mass error between -5 and 5 ppm for tryptophan without Easy-IC, and -2 and 2 with Easy-IC lock-mass correction. Tryptophan retention time should not deviate by more than ±0.3 min (HILIC) or ±0.1 min (RPLC) from the average, and the signal should be above 5E5 (HILIC) and 5E6 (RPLC). The chromatogram of all analytes in QC_SS_ and blanks were visually evaluated. System suitability was performed at least every 4^th^ batch. Unless batches were run consecutively, the systems were conditioned with four or seven QC_pool_ injections for RPLC and HILIC, respectively. During sample acquisition, each sample was evaluated (sample release) and each run was evaluated (analytical run release) before the injection plate was discarded. For sample release, the ISTD signals were monitored in each injection to verify the successfully executed injection cycle and identify possible errors and drops in sensitivity over the runs. The sensitivity and retention time stability were evaluated with leucine-ISTD and tryptophan-ISTD on HILIC and RPLC, respectively. If the sensitivity of the ISTD fell below approximately 50% compared with immediately after preventive maintenance, then a system suitability test was run at the end of the batch and preventive was maintenance performed. Analytical run release was performed by monitoring selected endogenous metabolites in the QC_pool_, QC_H_, QC_L_, and blank injections. Acceptance criteria were as follows (qualitative, not numeric): upregulation in QC_H_ (compared with QC_pool_), downregulation in QC_L_ (compared with QC_pool_), and absence in method blanks. Evaluation was made for tryptophan, glycocholate, caffein, arginine, and methionine.


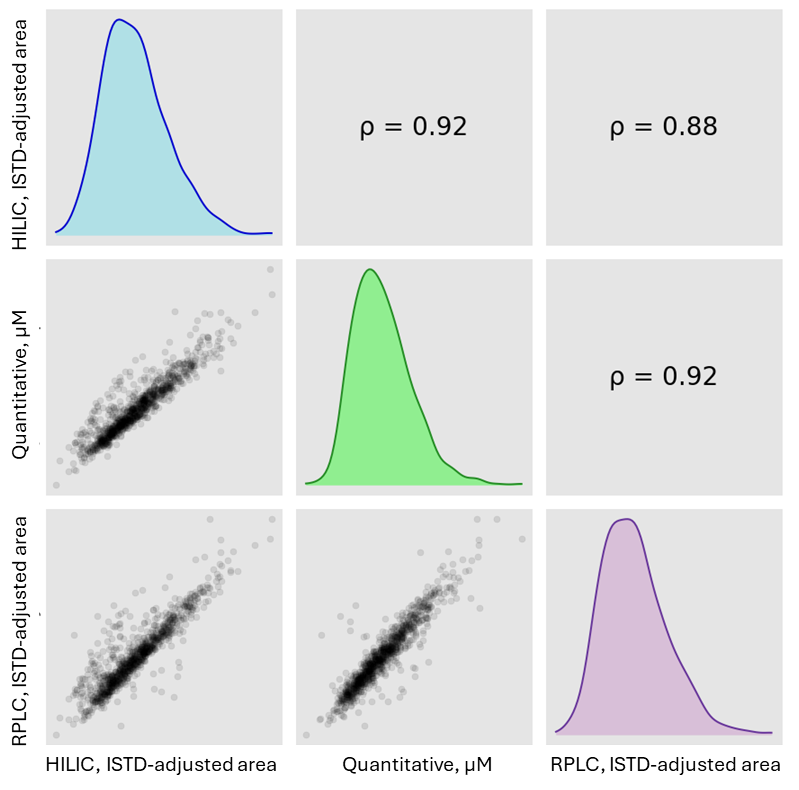


*Figure SI1:* *Pairplot of methionine results from each sample (N=1000) evaluated on three different methods, below the diagonal are scatterplots where hydrophilic interaction liquid chromatography (HILIC) and Reversed-phase liquid chromatography (RPLC) are presented as internal standard (ISTD)-adjusted areas, and targeted results in µM. The diagonal row presents kernel density estimate plots for HILIC (blue), targeted (green), and RPLC (purple) measurements. To the top right of the diagonal are given Spearman’s rho correlation coefficient of measurements.*

*Table SI1: Sample list for an untargeted hydrophilic interaction liquid chromatography – negative electrospray ionization – high-resolution mass spectrometry (HILIC-ESI^-^-HRMS) analytical run. The run order and positions are identical for the reversed-phase liquid chromatography method. The “Type” column is for illustration in this table and is not used in the vendor software.*

| Type | File Name | Position | Inj Vol |
| --- | --- | --- | --- |
| Conditioning | ANBU20_CQC01 | B:B1 | 3 |
| Conditioning | ANBU20_CQC02 | B:B1 | 3 |
| Conditioning | ANBU20_CQC03 | B:B1 | 3 |
| Conditioning | ANBU20_CQC04 | B:B1 | 3 |
| Conditioning | ANBU20_CQC05 | B:B1 | 3 |
| Conditioning | ANBU20_CQC06 | B:B1 | 3 |
| QC_pool_ | ANBU20_QCpool01 | R:b1 | 3 |
| QC_pool_ | ANBU20_QCpool02 | R:c1 | 3 |
| Sample | ANBU20_T0837 | R:d1 | 3 |
| Sample | ANBU20_T0838 | R:e1 | 3 |
| Sample | ANBU20_T0839 | R:f1 | 3 |
| Sample | ANBU20_T0840 | R:g1 | 3 |
| Sample | ANBU20_T0841 | R:h1 | 3 |
| Sample | ANBU20_T0842 | R:a2 | 3 |
| QC_pool_ | ANBU20_QCpool03 | R:b2 | 3 |
| QC_H_ | ANBU20_QCH01 | R:c2 | 3 |
| Sample | ANBU20_T0843 | R:d2 | 3 |
| Sample | ANBU20_T0844 | R:e2 | 3 |
| Sample | ANBU20_T0845 | R:f2 | 3 |
| Sample | ANBU20_T0846 | R:g2 | 3 |
| Sample | ANBU20_T0847 | R:h2 | 3 |
| QC_pool_ | ANBU20_QCpool04 | R:a3 | 3 |
| Sample | ANBU20_T0848 | R:b3 | 3 |
| Sample | ANBU20_T0849 | R:c3 | 3 |
| Sample | ANBU20_T0850 | R:d3 | 3 |
| QC_L_ | ANBU20_QCL01 | R:e3 | 3 |
| Sample | ANBU20_T0851 | R:f3 | 3 |
| Sample | ANBU20_T0852 | R:g3 | 3 |
| QC_pool_ | ANBU20_QCpool05 | R:h3 | 3 |
| Sample | ANBU20_T0853 | R:a4 | 3 |
| Sample | ANBU20_T0854 | R:b4 | 3 |
| Sample | ANBU20_T0855 | R:c4 | 3 |
| Sample | ANBU20_T0856 | R:d4 | 3 |
| Sample | ANBU20_T0857 | R:e4 | 3 |
| Sample | ANBU20_T0858 | R:f4 | 3 |
| QC_pool_ | ANBU20_QCpool06 | R:g4 | 3 |
| Sample | ANBU20_T0859 | R:h4 | 3 |
| Sample | ANBU20_T0860 | R:a5 | 3 |
| Sample | ANBU20_T0861 | R:b5 | 3 |
| Sample | ANBU20_T0862 | R:c5 | 3 |
| Sample | ANBU20_T0863 | R:d5 | 3 |
| QC_H_ | ANBU20_QCH02 | R:e5 | 3 |
| QC_pool_ | ANBU20_QCpool07 | R:f5 | 3 |
| Sample | ANBU20_T0864 | R:g5 | 3 |
| Sample | ANBU20_T0865 | R:h5 | 3 |
| Sample | ANBU20_T0866 | R:a6 | 3 |
| Sample | ANBU20_T0867 | R:b6 | 3 |
| Sample | ANBU20_T0868 | R:c6 | 3 |
| Sample | ANBU20_T0869 | R:d6 | 3 |
| QC_pool_ | ANBU20_QCpool08 | R:e6 | 3 |
| Sample | ANBU20_T0870 | R:f6 | 3 |
| Sample | ANBU20_T0871 | R:g6 | 3 |
| Sample | ANBU20_T0872 | R:h6 | 3 |
| Sample | ANBU20_T0873 | R:a7 | 3 |
| QC_L_ | ANBU20_QCL02 | R:b7 | 3 |
| Sample | ANBU20_T0874 | R:c7 | 3 |
| QC_pool_ | ANBU20_QCpool09 | R:d7 | 3 |
| Sample | ANBU20_T0875 | R:e7 | 3 |
| Sample | ANBU20_T0876 | R:f7 | 3 |
| Sample | ANBU20_T0877 | R:g7 | 3 |
| Sample | ANBU20_T0878 | R:h7 | 3 |
| Sample | ANBU20_T0879 | R:a8 | 3 |
| Sample | ANBU20_T0880 | R:b8 | 3 |
| QC_pool_ | ANBU20_QCpool10 | R:c8 | 3 |
| QC_Batchpool_ | ANBU20_Bpool | R:d8 | 3 |
| QC_SRM1950_ | ANBU20_SRM1950 | R:e8 | 3 |
| QC_Mblank_ | ANBU20_Mblank | R:a1 | 3 |
| QC_ISblank_ | ANBU20_Isblank | R:g12 | 3 |
| QC_noIS_ | ANBU20_QCnoIS | R:h12 | 3 |
| QC_pool_ | ANBU20_QCpool11 | R:f8 | 3 |
| QC_pool_ | ANBU20_QCpool12 | R:g8 | 3 |
| ExtraQC | ANBU20_Linearity_0_5 | R:g8 | 0.5 |
| ExtraQC | ANBU20_Linearity_1 | R:g8 | 1 |
| ExtraQC | ANBU20_Linearity_2 | R:g8 | 2 |
| ExtraQC | ANBU20_Linearity_4 | R:g8 | 4 |
| ExtraQC | ANBU20_Linearity_5 | R:g8 | 5 |
| ExtraQC | ANBU20_Linearity_6 | R:g8 | 6 |
| ExtraQC | ANBU20_Linearity_7 | R:g8 | 7 |

*Table SI2: Analytical results for investigated targets on evaluated metabolomics methods. Left column: Extracted Ion Chromatograms ±3ppm from the exact mass are presented in an allopurinol-positive (blank), allopurinol-negative (red), and blank sample (green), normalized to the highest value. NL shows highest peak (arbitrary units) in displayed chromatograms. The blank sample is not visible in any of the chromatograms. Levels refers to metabolite identification confidence. Right column: Ion trap MS/MS for each target, including information in the scan header of precursor ion that triggered the MS/MS and retention time. HILIC: hydrophilic interaction liquid chromatography, RPLC: reversed-phase liquid chromatography*

| Oxipurinol_HILIC (level 2)  Deduced from fragmentation and rt | Analytical drug target of allopurinol |
| --- | --- |
|  |  |
| Xanthine_HILIC (level 1)  Δ rt from library: 0.07 min | Endogenous metabolite marker |
|  |  |
| Allopurinol_HILIC (level 2)  Visual match with mzCloud | Analytical drug target of allopurinol |
|  |  |
| Hypoxanthine_HILIC (level 1)  Δ rt from library: 0.11 min | Endogenous metabolite marker |
|  |  |
| Allopurinol-1-riboside_HILIC (level 2)  Deduced from spectral similarity with allopurinol | Analytical drug target of allopurinol |
|  |  |
| Orotate_HILIC (level 1)  Δ rt from library: 0.31 min | Endogenous metabolite marker |
|  |  |
| Orotidine_ HILIC (level 2)  Deduced from spectral similarity with orotate | Endogenous metabolite marker |
|  |  |
| Uric acid_ HILIC (level 1)  Δ rt from library: 0.02 min | Endogenous metabolite marker |
|  |  |
| Oxipurinol_glycoside_HILIC (level 2)  Deduced from spectral similarity with oxipurinol | Analytical drug target of allopurinol |
|  |  |
| Uric acid_RP (level 3) | Endogenous metabolite marker |
|  | No IT-MS/MS recorded |
| Hypoxanthine/Allopurinol_RPLC (chimeric spectrum, identification level not defined) | Analytical drug target of allopurinol/Endogenous metabolite marker |
|  |  |
| Xanthine/Oxipurinol_RPLC (chimeric spectrum, identification level not defined) | Analytical drug target of allopurinol/Endogenous metabolite marker |
|  | No IT-MS/MS recorded |
| Febuxostat_RPLC (level 2)  Visual match with mzCloud |  |
|  |  |
| Febuxostat-M (-OH)_RPLC (level 2)  Deduced from febuxostat identification |  |
|  |  |

References

1. Sommer U, Koal T, Peck A. Application note: Standardized Targeted Metabolomics Using the BIOCRATES MxP Quant 500 Kit on the ACQUITY UPLC I-Class PLUS and Xevo TQ-XS Mass Spectrometer. 2019.

2. Zararsiz GE, Lintelmann J, Cecil A, Kirwan J, Poschet G, Gegner HM, et al. Interlaboratory comparison of standardised metabolomics and lipidomics analyses in human and rodent blood using the MxP® Quant 500 kit. bioRxiv [Internet]. 2024 Jan 1;2024.11.13.619447. Available from: http://biorxiv.org/content/early/2024/11/14/2024.11.13.619447.abstract

3. Grijseels S, Vasskog T, Heinsvig PJ, Myhre TN, Hansen T, Mardal M. Validation of two LC–HRMS methods for large-scale untargeted metabolomics of serum samples: Strategy to establish method fitness-for-purpose. J Chromatogr A. 2024;1732(May).

4. Zheng F, Zhao X, Zeng Z, Wang L, Lv W, Wang Q, et al. Development of a plasma pseudotargeted metabolomics method based on ultra-high-performance liquid chromatography–mass spectrometry. Nat Protoc [Internet]. 2020;15(8):2519–37. Available from: http://dx.doi.org/10.1038/s41596-020-0341-5
